# Prevalence and Clinical Impact of Obstructive Sleep Apnea in Patients with Severe Aortic Stenosis Undergoing Valve Replacement

**DOI:** 10.3390/biomedicines13051252

**Published:** 2025-05-21

**Authors:** Hilary Miranda-Mendoza, Luis M. Amezcua-Guerra, Gustavo Rojas-Velasco, Daniel Manzur-Sandoval, Jennifer Escobar-Alvarado, Luis Chávez-Sánchez, Wendy G. Vázquez-González, Laura L. Rodríguez Chávez, Humberto Martínez Hernández, Malinalli Brianza-Padilla

**Affiliations:** 1 Departamento de Inmunología, Instituto Nacional de Cardiología Ignacio Chávez, Mexico City 14080, Mexico; hilary.miranda001@gmail.com (H.M.-M.); lmamezcuag@gmail.com (L.M.A.-G.); 2 Red MEDICI, Facultad de Estudios Superiores Iztacala, Universidad Nacional Autónoma de México, Tlalnepantla 54090, Mexico; 3 Programa de Maestría en Ciencias Químico Biológicas, Escuela Nacional de Ciencias Biológicas, Instituto Politécnico Nacional, Mexico City 07738, Mexico; 4 Unidad de Terapia Intensiva Cardiovascular, Instituto Nacional de Cardiología Ignacio Chávez, Mexico City 14080, Mexico; gustavorojas08@gmail.com (G.R.-V.); drdanielmanzur@gmail.com (D.M.-S.); 5 Laboratorio de Sueño, Unidad de Investigación UNAM-INCICH, Mexico City 14080, Mexico; jenniferescobar15@gmail.com; 6 Unidad de Investigación Médica en Enfermedades Metabólicas del Hospital de Cardiología, Centro Médico Nacional Siglo XXI, Instituto Mexicano del Seguro Social, Mexico City 06720, Mexico; luis_chz@hotmail.com (L.C.-S.); wendisya@live.com.mx (W.G.V.-G.); 7 Unidad de Investigación Médica en Inmunología del Hospital de Pediatría, Centro Médico Nacional Siglo XXI, Instituto Mexicano del Seguro Social, Mexico City 06720, Mexico; 8 Laboratorio Central de Epidemiología, División de Laboratorios Especializados, Centro Médico Nacional la Raza, Instituto Mexicano del Seguro Social, Mexico City 06720, Mexico; 9 Cardiología Adultos, Piso 3, Instituto Nacional de Cardiología Ignacio Chávez, Mexico City 14080, Mexico; lauraleticiar@gmail.com; 10 Departamento de Cirugía, Instituto Nacional de Cardiología Ignacio Chávez, Mexico City 14080, Mexico; humbertomartinez@hotmail.com

**Keywords:** obstructive sleep apnea, aortic stenosis, postoperative recovery

## Abstract

**Background/Objectives**: Aortic stenosis (AS) is the most prevalent valvular disease among older adults. Although obstructive sleep apnea (OSA) has been linked to adverse cardiovascular outcomes, its specific impact on patients with severe AS remains unclear. This study aimed to determine the prevalence of OSA and its influence on postoperative recovery following aortic valve replacement. **Methods**: A prospective case-control study was conducted at the Instituto Nacional de Cardiología Ignacio Chávez. Patients aged 40–80 years with echocardiographically confirmed severe AS were categorized into groups with and without OSA, based on respiratory polygraphy (Apnea–Hypopnea Index [AHI] threshold of >15 events per hour). Clinical, biochemical, echocardiographic, body composition, and hemodynamic parameters were assessed. Daytime sleepiness and sleep quality were evaluated using validated questionnaires. Inflammatory biomarkers were also analyzed. This study was approved by the institutional ethics committee. **Results**: Of the 30 patients included, 66.6% were diagnosed with OSA. Compared to non-OSA patients, those with OSA had a higher left ventricular mass index (160 vs. 108; *p* = 0.001), greater postoperative increases in central venous pressure [8 (8–10) vs. 8 (6–8); *p* = 0.037], and lower mixed venous oxygen saturation within the first 24 h (69.2 vs. 76; *p* = 0.027). OSA patients also had longer hospital stays (11 vs. 8 days; *p* = 0.014). Trends toward a heightened subclinical inflammatory state were noted in the OSA group. **Conclusions**: OSA is frequent and underdiagnosed in patients with severe AS and is associated with more complicated postoperative recovery. Systematic OSA screening is recommended for candidates undergoing aortic valve surgery.

## 1. Introduction

Aortic stenosis (AS) is the most common valvular heart disease in the adult population, with its prevalence increasing significantly with age. In 2017, there were ~12.6 million cases of calcific AS, increased by 124% from 1990, with 102,700 AS-related deaths globally [1,2]. In high-income regions, the prevalence of calcific AS is notably higher. For instance, in 2019, Western Europe reported an age-standardized death rate of 4.05 per 100,000 persons due to AS [3]. Major risk factors include advanced age, male sex, dyslipidemia, and smoking [4,5,6,7]. AS typically follows a gradual and progressive course, characterized by increasing obstruction of the left ventricular (LV) outflow tract [8,9]. In response to this restriction, the heart undergoes compensatory concentric hypertrophy [10], which impairs diastolic function and hampers ventricular filling [11,12]. Beyond its hemodynamic consequences, progressive LV hypertrophy increases myocardial oxygen demand [13]. This mismatch between oxygen supply and demand, along with the compression of intramural coronary arteries, compromises myocardial perfusion and promotes ischemia [14].

In recent years, sleep-disordered breathing—particularly obstructive sleep apnea (OSA)—has emerged as a relevant contributor to the progression of AS. The prevalence of OSA in patients with AS has been reported to be as high as 35% [15], and its presence is associated with a marked increase in cardiovascular events, reaching up to 90% [16]. OSA is an underdiagnosed respiratory disorder characterized by recurrent episodes of partial or complete upper airway obstruction during sleep. These events result in intermittent hypoxemia, repetitive reoxygenation, nocturnal micro-arousals, sympathetic nervous system hyperactivation, and sleep fragmentation. Therefore, patients often experience non-restorative sleep, excessive daytime sleepiness, impaired quality of life, and increased cardiovascular risk [17].

In the setting of AS, coexisting OSA may exacerbate disease progression through mechanisms such as increased LV afterload [18], chronic nocturnal hypoxemia [19], and intrathoracic pressure fluctuations that exacerbate mechanical stress on the aortic valve [20]. These pathophysiological interactions highlight the clinical importance of timely OSA detection and management in patients with AS.

The objective of this study is to evaluate the association between OSA and AS in patients undergoing aortic valve replacement, with a special focus on its impact on clinical outcomes and postoperative recovery.

## 2. Materials and Methods

### 2.1. Patient Selection and Control Group

This prospective case-control study included patients aged 40 to 80 years who underwent surgical intervention at the Instituto Nacional de Cardiología Ignacio Chávez (INCICh) between March 2023 and October 2024, with a confirmed diagnosis of severe AS.

The case group comprised patients who underwent aortic valve replacement and met diagnostic criteria for AS based on the 2008 Mexican Clinical Practice Guidelines (CPG) [21], in addition to a diagnosis of OSA, defined by an Apnea–Hypopnea Index (AHI) > 15. The control group included patients with calcific AS undergoing surgical treatment at INCICh, without OSA (AHI ≤ 15).

Exclusion criteria included a history of acute myocardial infarction, presence of a pacemaker, non-valvular heart disease, autoimmune or neoplastic diseases, major surgery or blood transfusion within the preceding six months, systemic infections, or immunosuppression.

Data on concomitant medication use—including antihypertensive agents, antidiabetic drugs, statins, and antiplatelet therapy—were collected through a detailed review of each patient’s electronic medical records. Data collection was performed by a single reviewer and included both chronic outpatient prescriptions and medications administered during hospitalization. This allowed for accurate documentation of pharmacologic management of comorbid conditions, particularly those related to cardiovascular and metabolic risk.

All participants provided written informed consent. The study was approved by the Institutional Ethics Committee (registration number 23-1362), and all procedures adhered to the principles of the Declaration of Helsinki (2013) and applicable local regulations.

### 2.2. Diagnosis of Aortic Stenosis

Patients were initially evaluated in the valvular heart disease outpatient clinic at INCICh, presenting with symptoms suggestive of AS. Comprehensive transthoracic echocardiography was performed using a Vivid 7™ system (GE Healthcare, Chicago, IL, USA), following guidelines from the American Society of Echocardiography (ASE) and the European Association of Cardiovascular Imaging (EACVI) [22]. Evaluated parameters included aortic valve anatomy and hemodynamics (transvalvular gradient, residual valve area), pulmonary artery pressures, and left ventricular ejection fraction (LVEF), which was calculated using the modified Simpson’s method [23].

Left atrial area and contractility were also measured via multiple transthoracic approaches, including CW-Doppler waveform, jet diameter, and central jet width. AS severity was classified in accordance with current ACC/AHA guidelines. All included patients met criteria for severe AS (peak aortic jet velocity ≥ 4 m/s, mean gradient ≥ 40 mmHg, aortic valve area ≤ 0.6 cm^2^) [24].

### 2.3. Aortic Valve Replacement

All patients underwent median sternotomy, followed by an aortotomy. A St. Jude Masters mechanical prosthesis (St. Jude Medical Inc., St. Paul, MN, USA) was implanted using the “parachute” technique with self-reinforced sutures. Procedures were conducted under general anesthesia via upper T-inverted partial sternotomy with cardiopulmonary bypass support and controlled hypothermia.

In the control group (n = 9), six patients received a 21 mm St. Jude Masters prosthesis, and three received a 23 mm prosthesis. In the case group (n = 21), seven patients received a 19 mm, two a 21 mm, and twelve a 23 mm St. Jude Masters prosthesis.

### 2.4. Intensive Care Unit Stay

Postoperatively, all patients were admitted to the Intensive Care Unit (ICU) and received standard care, including advanced hemodynamic monitoring to assess cardiovascular function and tissue perfusion.

The hemodynamic variables recorded were cardiac output (CO), systemic vascular resistance index (SVRI), central venous pressure (CVP), mixed venous oxygen saturation (SvO_2_), arteriovenous oxygen difference (A-V O_2_ diff), and oxygen extraction ratio (O_2_ER%).

Functional status and surgical risk were assessed using the New York Heart Association (NYHA) classification [25], EUROSCORE II [26], and the Sequential Organ Failure Assessment (SOFA) score [27]. ICU length of stay was documented in complete days.

### 2.5. Laboratory Analyses

Following informed consent and prior to aortic valve replacement, 15 mL of peripheral venous blood was collected using Vacutainer tubes with clot activator gel (BD, Franklin Lakes, NJ, USA). Samples were centrifuged at 3500 rpm for 10 min at room temperature, and sera were aliquoted and stored at −80 °C.

Sera were thawed under standard conditions, and cytokine levels were measured using ELISA kits (FineTest) in accordance with manufacturer instructions. The following cytokines were measured: IL-6 (4.688–300 pg/mL), IL-1β (3.906–250 pg/mL), and IL-10 (7.813–500 pg/mL).

Monocytes were isolated using Histopaque-1077 (Sigma-Aldrich, St. Louis, MO, USA) following a standardized protocol. Blood samples were collected in EDTA tubes and processed within a maximum of two hours after extraction. The collected cells were washed with phosphate-buffered saline (PBS), and cell viability was assessed using trypan blue exclusion (0.4%) (Sigma-Aldrich, St. Louis, MO, USA).

For phenotypic characterization of monocytes, samples were stained with specific fluorochrome-conjugated antibodies. The cells were incubated with antibodies against CD14 (Abcam, Cambridge, UK), CD3 (Abcam, Cambridge, UK), and CD16 (Abcam, Cambridge, UK) for 20 min at 4 °C in the dark to prevent fluorochrome photodegradation. Readings were performed using a Cytek Aurora 5-laser spectral flow cytometer (Cytek Biosciences Inc., Fremont, CA, USA), applying optimized acquisition settings for the detection of the employed fluorochromes.

Additionally, the following biochemical and hematological parameters were measured using the Roche Cobas c701 automated analyzer (Roche Diagnostics, Mannheim, Germany): total cholesterol (<200 mg/dL), HDL (≥60 mg/dL), LDL (<100–159 mg/dL), triglycerides (40–<150 mg/dL), glucose (74–106 mg/dL), creatinine (0.5–1.2 mg/dL), albumin (3.9–4.9 g/dL), leukocytes (3.84–9.79 × 10^3^/μL), lymphocytes (0.99–3.24 × 10^3^/μL), monocytes (0.19–0.71 × 10^3^/μL), platelets (150–450 × 10^3^/μL), neutrophils (1.71–6.48 × 10^3^/μL), high-sensitivity C-reactive protein (hsCRP; <5.0 mg/L), N-terminal pro–B-type natriuretic peptide (NT-proBNP; 5–162.4 pg/mL), and fibrinogen (1.90–5.13 g/L).

### 2.6. Sleep Quality Assessment

During the patients’ initial visit to the INCICh and after informed consent, validated questionnaires were administered to assess sleep quality. To ensure interpretative accuracy, versions validated for the Mexican population were used [28,29].

The Pittsburgh Sleep Quality Index (PSQI) is a self-administered questionnaire that evaluates sleep quality over the previous month. It comprises seven components: subjective sleep quality, sleep latency, sleep duration, habitual sleep efficiency, sleep disturbances, use of hypnotic medications, and daytime dysfunction. Each component is scored from 0 to 3, yielding a global score ranging from 0 to 21. A global score > 5 is considered indicative of poor sleep quality [30].

The Epworth Sleepiness Scale (ESS) is a questionnaire that measures the subjective likelihood of falling asleep in eight common daily situations. The total score ranges from 0 to 24, with higher scores indicating greater daytime sleepiness. Scores < 10 are considered normal, whereas higher values suggest the need for further medical evaluation [31].

To classify sleep duration, participants were categorized into three groups: short sleep (<6 h/night), normal sleep (7–8 h/night), and long sleep (>9 h/night).

### 2.7. Respiratory Polygraphy

All patients underwent overnight respiratory polygraphy using the Alice NightOne device (Philips Respironics, Murrysville, PA, USA). Studies with recording times < 240 min were excluded. The following parameters were calculated: AHI, the average number of apnea and hypopnea episodes divided by the total recording time (expressed in events/hour); Obstructive Apnea Index (OAI): the number of obstructive apnea events per hour of sleep; Oxygen Desaturation Index (ODI): the number of ≥4% oxygen desaturation events per hour of recording; minimum oxygen saturation (minimum SaO_2_): the lowest oxygen saturation value recorded during overnight monitoring.

Respiratory polygraphy recordings were acquired and analyzed manually by a single operator (E-A, J.), using a standardized scoring protocol.

### 2.8. Diagnosis of Obstructive Sleep Apnea

OSA was diagnosed based on the International Consensus Document on Obstructive Sleep Apnea [32], defined as AHI ≥ 15 events/hour, predominantly of obstructive type.

### 2.9. Somatometry

Body composition was assessed via multifrequency bioelectrical impedance analysis with the Seca mBCA Ultra device (Seca GmbH & Co. KG, Hamburg, Germany). This analysis included both segmental and whole-body measurements of the following variables: body weight (kg), body mass index (BMI, kg/m^2^), body fat percentage (%), phase angle (°), fat mass (%), lean mass (%), visceral fat (L), total body water (%), extracellular water (%), skeletal muscle mass (kg), basal metabolic rate (kcal/day), and waist-to-hip ratio (WHR).

### 2.10. Statistical Analysis

Normality was assessed with the Shapiro–Wilk test. Given the non-normal distribution of most variables, quantitative variables were expressed as medians with interquartile ranges (IQRs) and categorical variables as frequencies and percentages. Nonparametric tests were used: Mann–Whitney U test for two-group comparisons and Fisher’s exact test for categorical variables. Statistical significance was set at *p* < 0.05. Statistical analyses were performed using GraphPad Prism software version 9.4 (GraphPad Inc., La Jolla, CA, USA).

Additionally, a post hoc power analysis was conducted using G*Power 3.1 software (Heinrich Heine University, Düsseldorf, Germany) to assess the adequacy of the sample size (n = 30) for detecting clinically relevant differences across key outcome variables. Effect sizes (r) were derived from the observed *p*-values and subsequently converted to Chen’s d to classify the magnitude of difference.

## 3. Results

### 3.1. Clinical Characteristics of Patients at Hospital Admission (Pre-Surgery)

A total of 30 patients diagnosed with severe AS and scheduled for aortic valve replacement at the INCICh were enrolled in the study. Of these, 21 patients (66.6%) were diagnosed with OSA, while the remaining 9 patients (33.3%) did not present evidence of OSA. The median age in the OSA group was 62 years [59.5–64.5] compared to 61 years [54–65.5] in the non-OSA group. Patients with OSA exhibited lower oxygen saturation compared to the control group [94 (93–95) vs. 96 (94–96); *p* = 0.043]. Comorbidities were highly prevalent across the cohort, with a particularly elevated burden observed in the OSA group (Table 1).

### 3.2. Body Composition

Analysis of body composition revealed no significant differences between patients with and without OSA. The average waist-to-hip ratio was 0.98, neck circumference was 38.5 cm, and BMI was 27.2 kg/m^2^. Other parameters included a phase angle of 5°, body fat percentage of 35%, lean mass percentage of 64.8%, visceral fat volume of 3.1 L, total body water percentage of 47.7%, and extracellular water percentage of 21.4%.

### 3.3. Echocardiographic Parameters

In the echocardiographic evaluation, patients with OSA exhibited a significantly higher left ventricular mass index compared to those without OSA (160 g/m^2^ [130–176] vs. 108 g/m^2^ [84–116]; *p* = 0.001). Additionally, peak velocity in the left ventricular outflow tract was significantly reduced in the OSA group (0.75 m/s [0.67–0.98] vs. 0.9 m/s [0.85–1.11]; *p* = 0.036) (Table 2).

### 3.4. Sleep Quality

No significant differences were observed in sleep quality between groups. Both groups had a mean PSQI score of 7, indicating suboptimal sleep quality. The reported total sleep duration averaged approximately 7 h in both groups. Similarly, the mean score on the ESS was 6.5 in both groups, suggesting a comparable level of daytime sleepiness.

### 3.5. Respiratory Polygraphy

Respiratory polygraphy analysis confirmed the presence of significant sleep-disordered breathing in OSA patients. In this group, the AHI was 34.4 events per hour (20.8–47.2), predominantly composed of obstructive events. The total number of obstructive events was 148 (73–274), and the OAI was 21.6 (8.8–29.9). Details of respiratory polygraphy findings are presented in Table 3.

### 3.6. Laboratory Data

Most laboratory parameters did not differ significantly between groups. However, the atherogenic index was significantly higher in patients with OSA compared to those without OSA (2.8 [2.1–3.4] vs. 2.2 [1.4–2.6]; *p* = 0.018) Table 4.

### 3.7. Intensive Care Unit Stay

The duration of postoperative hospital stay was significantly longer in patients with OSA compared to those without OSA (11 days [8.5–16] vs. 8 days [6.5–10]; *p* = 0.014). No significant differences were found in cardiac risk scores such as the EUROSCORE II or in NYHA functional classification.

During ICU stay, patients with OSA showed a significant increase in central venous pressure (CVP), from a median of 8 mmHg (6–8) at admission to 8 mmHg (8–10) at 24 h postoperatively (*p* = 0.037). Furthermore, within the first 24 h after surgery, a significant reduction in mixed venous oxygen saturation (%SvO_2_) was observed in the OSA group (from 76% [68.5–83] to 69.2% [63–74.1]; *p* = 0.027). Correspondingly, the oxygen extraction ratio (%O_2_ER) increased from 24% (19–29) at baseline to 29% (24.5–31) at 24 h postoperatively in the OSA group (Table 5). The analysis also included the duration of invasive mechanical ventilation (IMV), with a mean of 1.3 ± 1.1 days in the OSA group and 0.8 ± 0.4 days in the non-OSA group.

## 4. Discussion

This study evaluated the prevalence and clinical impact of OSA in patients with severe AS undergoing aortic valve replacement, with particular attention to postoperative outcomes. Our findings revealed a high prevalence of previously undiagnosed OSA (66.6%) in patients with severe AS, aligning with previous studies reporting prevalence rates between 35% and 70% in this population [33,34]. The clinical relevance of these findings relies in the fact that symptoms attributable to OSA, such as fatigue, dyspnea, or poor sleep quality, may be erroneously ascribed to the valvular pathology or to aging itself, leading to misdiagnosis.

One of the notable findings of this study was the statistically significant difference in postoperative hospital stay observed in patients with OSA compared to those without OSA (11 vs. 8 days), suggesting that OSA confers increased postoperative hemodynamic vulnerability and a heightened inflammatory burden. This may be attributed to the accumulative effects of intermittent hypoxemia, sympathetic overactivation, and oxidative stress—hallmarks of OSA—that have been implicated in maladaptive left ventricular remodeling [35,36]. Notably, even in the absence of overt heart failure, these pathophysiological mechanisms may promote myocardial hypertrophy and diastolic dysfunction [10,11,37]. The prolonged stay in the ICU has meaningful clinical and prognostic implications. Extended ICU stays are associated with a greater risk of nosocomial infections, increased healthcare costs due to the use of specialized personnel and advanced technologies, and a higher likelihood of developing post-intensive care syndrome (PICS), which encompasses long-term cognitive, psychological, and physical impairments [38].

In line with this mechanistic hypothesis, patients with OSA showed increased left ventricular mass and elevated NT-proBNP levels (approximately threefold higher than in non-OSA patients), suggesting subclinical hemodynamic overload. Although statistical significance was not reached for NT-proBNP, the observed trends are clinically relevant and consistent with the hypothesis that OSA acts as an independent cardiovascular stressor in patients with AS, potentially accelerating disease progression.

From a hemodynamic perspective, patients with OSA exhibited a significant postoperative increase in CVP and a decrease in %SvO_2_ within the first 24 h. These changes indicate reduced oxygen delivery efficiency and increase metabolic demand, likely reflecting limited cardiovascular functional reserve. Intermittent hypoxia may further exacerbate these changes by inducing endothelial dysfunction, promoting sympathetic activation, and impairing microvascular autoregulation [36].

Regarding the observed reduction in %SvO_2_ during the first 24 h after surgery in the OSA group, it is important to note that most patients were extubated between 12 and 18 h postoperatively, according to institutional weaning protocols. All extubations were performed under conditions of hemodynamic stability and adequate gas exchange. Additionally, the mean BMI in the OSA group was 27.2 kg/m^2^, consistent with an overweight profile. Both delayed extubation and increased BMI are known to influence oxygen transport and utilization and may partially explain the decline in %SvO_2_ observed postoperatively.

Our findings are consistent with large-scale epidemiological studies linking OSA to an increased risk of cardiovascular complications, including acute myocardial infarction, heart failure, and mortality [39]. The Sleep Heart Health Study reported a 35% increased incidence of coronary artery disease in individuals with moderate-to-severe OSA [40], while the Multi-Ethnic Study of Atherosclerosis identified a 2.4-fold increase in cardiovascular events and mortality among OSA patients [15].

Regarding inflammation, although no significant differences in serum cytokines were found between groups, patients with OSA exhibited elevated hsCRP levels and a trend toward a higher proportion of proinflammatory CD14^+^/CD16^+^ monocytes. These findings support the concept of OSA as a low-grade inflammatory condition that contributes to the development and progression of cardiovascular disease [41].

An important methodological consideration is the limited sensitivity of self-reported tools for detecting sleep-disordered breathing. In this study, both OSA and non-OSA groups showed similarly elevated scores on the PSQI and ESS, highlighting the limited discriminatory power of these instruments in this specific patient population. Recent studies have questioned the sensitivity of these subjective measures, particularly in older adults and patients with AS [42,43,44]. Despite perceptions of poor sleep quality, the correlation between subjective symptoms and objective findings, such as the AHI, is often weak. This finding reinforces the need for objective sleep assessments, such as respiratory polygraphy or polysomnography, especially in patients with valvular heart disease being evaluated for surgery. Among these methods, respiratory polygraph is a safe and effective diagnostic tool for OSA in appropriately selected patients. Although it has certain limitations compared to polysomnography—most notably the inability to detect microarousals due to the absence of electroencephalographic monitoring—it offers considerable advantages in terms of accessibility, convenience, and clinical utility. While respiratory polygraphy may underestimate the frequency of hypopneas, polysomnography requires an overnight hospital stay in a fully equipped sleep laboratory. In addition, the lower cost and easy availability of polygraphy position it as a practical diagnostic tool for at-risk populations in real-world clinical settings [45].

It is worth noting that the most common comorbidities in this cohort—diabetes mellitus and arterial hypertension—were adequately controlled from both clinical and biochemical standpoints. In addition, anthropometric parameters such as BMI, visceral fat, and lean mass indicated a population with overweight status, which may further contribute to the observed cardiovascular risk.

Several limitations of this study should be acknowledged. First, this was a single-center investigation with a relatively small sample size and unbalanced group distribution, which may limit the generalizability of the findings. Nonetheless, the implementation of a standardized clinical protocol, objective sleep diagnostics, and comprehensive echocardiographic and biochemical assessments enhances the internal validity of the results. Second, although the primary analysis focused on hospital stay duration and selected hemodynamic parameters, other clinically relevant outcomes—such as postoperative mortality, arrhythmias, and respiratory complications—were not predefined endpoints. While no in-hospital deaths occurred in either group, the low frequency of arrhythmic and respiratory events limited the feasibility of formal statistical analysis. Future multicenter studies with larger, more balanced cohorts and extended follow-up are warranted to validate these preliminary findings and provide a more comprehensive evaluation of the perioperative impact of obstructive sleep apnea in patients undergoing aortic valve replacement.

In conclusion, our findings suggest that OSA is a prevalent and underrecognized comorbidity in patients with severe AS that negatively affects postoperative recovery following valve replacement. The presence of OSA was associated with prolonged hospital stays, hemodynamic overload, and reduced tissue oxygenation efficiency, even in the absence of overt clinical symptoms. Finally, these findings advocate for systematic preoperative screening for OSA in patients with severe AS.

## Figures and Tables

**Table 1:** Clinical characteristics of patients at hospital admission.

	OSA n = 21	No OSA n = 9	*p*-Value
Male sex, n (%)	14 (66.6)	4 (44.4)	0.418
Age, median (IQR)	62 (59.5–64.5)	61 (54–65.5)	0.696
Diabetes n, (%)	7 (33.3)	3 (33.3)	>0.999
Hypertension, n (%)	12 (57.1)	3 (33.3)	0.427
Hypothyroidism, n (%)	1 (4.7)	0 (0)	>0.999
Smoking, n (%)	7 (33.3)	0 (0)	0.071
Alcohol use, n (%)	3 (14.3)	0 (0)	0.534
Heart rate, median (IQR) (bpm)	72 (69–76)	70 (62.5–81.5)	0.569
Systolic BP, median (IQR) (mmHg)	114 (102.5–121)	110 (103.5–117.5)	0.663
Diastolic BP, median (IQR) (mmHg)	70 (64–74.5)	68 (60–71.5)	0.364
Respiratory rate, median (IQR)	18 (16–18)	18 (16–18)	0.913
O_2_ saturation, median (IQR) (%)	94 (93–95)	96 (94–96)	0.043

Categorical variables are presented as percentages, and continuous variables are presented as medians with interquartile ranges (IQRs). Comparisons between groups were made using Fisher’s exact test for categorical variables and the Mann–Whitney U test for continuous variables. A *p*-value < 0.05 was considered statistically significant.

**Table 2:** Echocardiographic parameters of patients with and without obstructive sleep apnea.

	OSA n = 21	No OSA n = 9	*p*-Value
LVEF %, median (IQR)	52 (32–61)	57.2 (45.5–68)	0.330
Valve area cm^2^/m^2^, median (IQR)	0.57 (0.47–0.83)	0.6 (0.4–0.68)	0.837
Maximum gradient mmHg, median (IQR)	102 (73.3–131.4)	82 (74–140)	0.936
Mean gradient mmHg, median (IQR)	61 (20–84.3)	54 (43.5–85)	0.973
Peak transvalvular velocity m/s, median (IQR)	4.9 (4.2–5.7)	4.5 (4.3–5.9)	0.972
LVOT peak velocity m/s, median (IQR)	0.75 (0.67–0.98)	0.9 (0.85–1.11)	0.036
LV mass gr/m^2^, median (IQR)	160 (130–176)	108 (84–116)	0.001
Aortic diameter cm, median (IQR)	3.1 (2.8–3.9)	3.4 (2.8–3.6)	0.885
Aortic strain m/s, median (IQR)	1.2 (0.89–1.4)	1.5 (1.3–2)	0.107

Data are presented as medians with interquartile ranges (IQRs). Differences between groups were assessed using the Mann–Whitney U test. A *p*-value < 0.05 was considered statistically significant. Abbreviations: LVEF, left ventricular ejection fraction; LVOT, left ventricular outflow tract.

**Table 3:** Respiratory polygraphy parameters.

	OSA n = 21	No OSA n = 9	*p*-Value
AHI, median (IQR)	34.4 (20.8–47.2)	9.4 (3.8–13.7)	<0.001
Obstructive events, median (IQR)	148 (73–274)	21.8 (1–28.8)	<0.001
Hypopneas, median (IQR)	79 (62.5–136	33 (12–87)	0.022
Total events, median (IQR)	290 (168–390)	82 (31.5–116)	<0.001
Max event duration, median (IQR)	59 (53–66)	49.0 (38–59)	0.041
OAI, median (IQR)	21.6 (8.8–29.9)	1.2 (0.4–4.8)	<0.001
CAI, median (IQR)	0 (0–0.7)	0 (0–0)	0.195
ODI, median (IQR)	53.3 (33.5–62.6)	18.5 (5.3–28.8)7	<0.001
SaO_2_ < 90%, median (IQR)	52 (28.4–77.3)	53.4 (19–96.7)	0.763
SaO_2_ < 85%, median (IQR)	13 (0.8–24.3)	0.3 (0.05–54.5)	0.503
Mean heart, median (IQR) (bpm)	64.9 (58.2–71.9	65.1 (55–70.3)	0.696

Data are presented as medians with interquartile ranges (IQRs). Differences between groups were analyzed using the Mann–Whitney U test. A *p*-value < 0.05 was considered statistically significant. Abbreviations: AHI, Apnea–Hypopnea Index; OAI, Obstructive Apnea Index; CAI, Central Apnea Index; ODI, Oxygen Desaturation Index; SaO_2_, oxygen saturation.

**Table 4:** Laboratory data.

	OSA n = 21	No OSA n = 9	*p*-Value
Total cholesterol (mg/dL)	160 (125–205)	151 (132–187)	0.885
HDL mg/dL	38 (33.5–46.5)	40 (37.8–55.6)	0.283
LDL mg/dL	104 (72.6–142.5)	96.3 (74.9–115.5)	0.541
Triglycerides mg/dL	124 (102–155)	129 (113–172)	0.616
Atherogenic index (TC/HDL)	2.8 (2.1–3.4)	2.2 (1.4–2.6)	0.018
NT-proBNP (pg/mL)	1078 (540–3043)	427 (199–1922)	0.117
Glucose (mg/dL)	98.9 (95.9–105.5)	98.6 (88.7–106)	0.648
Creatinine (mg/dL)	1.03 (0.79–1.14)	0.87 (068–1.07	0.197
HbA1c (mmol/mol)	6.3 (5.6–6.5)	5.9 (5.3–5.9)	0.594
Albumin (g/dL)	3.1 (1–3.8)	2.1 (0–3.6)	0.282
Fibrinogen (g/L)	2.8 (2.4–3)	2.3 (1.6–2.9)	0.174
hsCRP	6.5 (0.8–51.6)	2.6 (1.6–6.4)	0.628
Leukocytes (×10^3^/μL)	6.1 (5.2–8.7)	6.4 (5.6–8.2)	0.722
% Lymphocytes	34.7 (28.9–39.1)	33.5 (21.4–47.6)	0.867
% Monocytes	8.4 (7.5–9.5)	7.8 (5.6–8.8)	0.320
% CD14+	72.9 (65–79.4)	71 (47–85.7)	>0.999
% CD14+/CD16+	18.5 (15.1–26.6)	22.7 (11.1–31.4)	>0.999
% CD16+	2.7 (1.5–5.4)	4.3 (1.6–15.8)	0.564
IL-6 (pg/mL)	9.3 (7.9–12.4)	10.7 (5.8–14.5)	0.935
IL-10 (pg/mL)	8.8 (4.6–29.2)	15 (5.8–67.5)	0.449
IL-1β (pg/mL)	43.3 (7.6–107)	46.8 (9–99.6)	0.991

Data are presented as medians and interquartile ranges (IQRs). Differences between groups were analyzed using the Mann–Whitney U test. A *p*-value < 0.05 was considered statistically significant.

**Table 5:** Hemodynamic and prognostic parameters in ICU.

	OSA			No OSA		
	Admission n = 21	24 h n = 21	*p*-Value	Admission n = 9	24 h n = 9	*p*-Value
CO (L/min)	3.4 (2.8–4.2)	3 (2.6–3.4)	0.701	3.3 (2.6–4.5)	3.2 (2.5–3.8)	0.679
CI (L/min/m^2^)	1.9 (1.4–2.5)	1.7 (1.4–2.2)	0.676	1.8 (1.3–2.7)	1.8 (1.4–2.4)	>0.999
CVP (mmHg)	8 (6–8)	8 (8–10)	0.037	8 (5.5–12)	8 (8–10)	0.781
SVRS (dynes.s/cm^5^/m^2^)	2431 (1971–3345)	2607 (2328–3616)	0.562	2345 (1877–3678)	2759 (2296–3295)	0.742
SvO_2_ (%)	76 (68.5–83)	69.2 (63–74.1)	0.027	70 (61.5–81.5)	67 (64.1–73.5)	0.312
A-V O_2_ (mL/100 mL)	3.7 (2.9–4.3)	4.05 (3.4–4.4)	0.647	3.9 (3.1–5.8)	4 (3.8–4.8)	>0.999
O_2_ER (%)	24 (19–29)	29 (24.5–31)	0.038	28 (17.5–38.5)	32 (29–33.6)	0.406
Lactate (mmol/L)	2 (1.1–3.3)	1.8 (1.5–2.8)	0.972	2.3 (1.3–4)	2 (1.5–2.1)	0.210
Capillary refill (s)	3 (2–3)	2 (2–3)	0.500	2 (2–3)	2 (2–3)	>0.999
SOFA score	3 (3–5)	2 (2–5)	0.040	4 (3–5)	4 (2–5)	0.578

Data are expressed as medians with interquartile ranges (IQRs). Differences between groups were analyzed using the Mann–Whitney U test. A *p*-value < 0.05 was considered statistically significant. Abbreviations: CO, cardiac output; CI, cardiac index; CVP, central venous pressure; SVRI, systemic vascular resistance index; SvO_2_, mixed venous oxygen saturation; A–V O_2_ diff, arteriovenous oxygen difference; O_2_ER, oxygen extraction ratio; SOFA, Sequential Organ Failure Assessment score.

## Data Availability

The data presented in this study are available on reasonable request from the corresponding author. The data are not publicly available due to ongoing related projects, as the investigators prefer to share them upon request to preserve academic priority for future analyses currently under development.
